# Brain connectivity and metacognition in persons with subjective cognitive decline (COSCODE): rationale and study design

**DOI:** 10.1186/s13195-021-00846-z

**Published:** 2021-05-25

**Authors:** Federica Ribaldi, Christian Chicherio, Daniele Altomare, Marta Martins, Szymon Tomczyk, Ileana Jelescu, Enrique Maturana, Max Scheffler, Sven Haller, Karl-Olof Lövblad, Michela Pievani, Valentina Garibotto, Matthias Kliegel, Giovanni B. Frisoni

**Affiliations:** 1 Laboratory of Neuroimaging of Aging (LANVIE), University of Geneva, Geneva, Switzerland; 2 Geneva Memory Center, Department of Rehabilitation and Geriatrics, Geneva University Hospitals, Geneva, Switzerland; 3 Center for Interdisciplinary Study of Gerontology and Vulnerability (CIGEV), University of Geneva, Geneva, Switzerland; 4 CIBM Center for Biomedical Imaging, Lausanne, Switzerland; 5 Animal Imaging and Technology, Ecole Polytechnique Fédérale de Lausanne, Lausanne, Switzerland; 6 Department of Radiology, Geneva University Hospitals, Geneva, Switzerland; 7 Division of Radiology, Geneva University Hospitals, Geneva, Switzerland; 8 CIMC - Centre d’Imagerie Médicale de Cornavin, Geneva, Switzerland; 9 Department of Surgical Sciences, Radiology, Uppsala University, Uppsala, Sweden; 10 Faculty of Medicine of the University of Geneva, Geneva, Switzerland; 11 Department of Radiology, Beijing Tiantan Hospital, Capital Medical University, Beijing, 100070 P. R. China; 12 Neurodiagnostic and Neurointerventional Division, Diagnostic Department, Geneva University Hospitals, Geneva, Switzerland; 13 Laboratory of Alzheimer’s Neuroimaging and Epidemiology (LANE), IRCCS Istituto Centro San Giovanni di Dio Fatebenefratelli, Brescia, Italy; 14 Laboratory of Neuroimaging and Innovative Molecular Tracers (NIMTlab), Geneva University Neurocenter and Faculty of Medicine, University of Geneva, Geneva, Switzerland; 15 Division of Nuclear Medicine and Molecular Imaging, Diagnostic Department, Geneva University Hospitals, Geneva, Switzerland; 16 Cognitive Aging Lab, Faculty of Psychology and Educational Sciences, University of Geneva, Geneva, Switzerland

**Keywords:** Subjective cognitive decline, Metacognition, Connectivity, Alzheimer’s disease biomarkers

## Abstract

**Background:**

Subjective cognitive decline (SCD) is the subjective perception of a decline in memory and/or other cognitive functions in the absence of objective evidence. Some SCD individuals however may suffer from very early stages of neurodegenerative diseases (such as Alzheimer’s disease, AD), minor psychiatric conditions, neurological, and/or somatic comorbidities. Even if a theoretical framework has been established, the etiology of SCD remains far from elucidated. Clinical observations recently lead to the hypothesis that individuals with incipient AD may have overestimated metacognitive judgements of their own cognitive performance, while those with psychiatric disorders typically present underestimated metacognitive judgements. Moreover, brain connectivity changes are known correlates of AD and psychiatric conditions and might be used as biomarkers to discriminate SCD individuals of different etiologies. The aim of the COSCODE study is to identify metacognition, connectivity, behavioral, and biomarker profiles associated with different etiologies of SCD. Here we present its rationale and study design.

**Methods:**

COSCODE is an observational, longitudinal (4 years), prospective clinical cohort study involving 120 SCD, and 80 control study participants (40 individuals with no cognitive impairment, and 40 living with mild cognitive impairment - MCI, or dementia due to AD), all of which will undergo diffusion magnetic resonance imaging (MRI) and functional magnetic resonance imaging (fMRI) as well as behavioral and biomarker assessments at baseline and after 1 and 2 years. Both hypothesis-driven and data-driven cluster analysis approaches will be used to identify SCD sub-types based on metacognition, connectivity, behavioral, and biomarker features.

**Conclusion:**

COSCODE will allow defining and interpreting the constellation of signs and symptoms associated with different etiologies of SCD, paving the way to the development of cost-effective risk assessment and prevention protocols.

## Background

Subjective cognitive decline (SCD) is a condition in which individuals perceive cognitive decline or impairment in the absence of objective evidence [1]. In the last 5–10 years, the condition has generated great interest in the fields of Alzheimer’s disease (AD) and dementia research since it has been shown to be associated with an increased risk of cognitive decline [1–4]. Advances in the fields of AD and dementia have resulted in increased awareness of brain health and prevention strategies. In this context, a growing number of SCD individuals seek advice at memory clinics, currently representing around 25% of a memory clinic’s population [5, 6]. However, studies on SCD are largely heterogeneous in terms of SCD operationalization, studied reference populations (memory clinic vs community-dwelling), biomarker assessment, and length of follow-up intervals. Consequently, there is variability in the reported prevalence of SCD itself and associated cognitive decline. Despite this variability, on average, 65% of cognitively unimpaired older individuals affirm SCD when asked [7, 8], and around 27% develop MCI and 14% dementia in the following 4 or more years [1, 3].

The SCD population is highly heterogeneous. Some SCD individuals may have preclinical AD, while others may be affected by psychopathological or psychiatric conditions (e.g., high levels of anxiety and depression, peculiar personality traits such as neuroticism [9–14]), or physical (vitamin D insufficiency or multimorbidity [15, 16]) or neurological comorbidities [17].

Currently, different worldwide initiatives try to understand which risk and protective factors are associated with cognitive decline and progression to dementia. Among them are large-scale European research projects including DELCODE, SCIENCE, and INSIGHT preAD [18–20]. The COSCODE project is inspired by these initiatives and aims to propose an alternative approach to identify *clinically relevant* SCD subtypes based on metacognition, connectivity, behavioral, and biomarker profiles. So far, studies on SCD were mainly focused on the association between SCD and AD biomarkers and/or depression and anxiety, but have rarely explored more deeply phenotyping of psychiatric features. One key novelty of the present approach is to systematically evaluate the role of metacognitive judgments in the context of SCD. The main hypothesis of COSCODE in this regard is based on the clinical observation that metacognitive judgments of an individuals’ own cognitive performance, rather than actual cognitive performance, may help to distinguish those likely to progress to MCI/dementia from others that do not. Specifically, individuals with preclinical AD tend to overestimate their actual cognitive performance (“my memory is poorer than it used to be, but it is adequate considering my age”), while individuals with depressive or neurotic traits tend to underestimate their actual cognitive performance (“my memory is much worse than what it used to be”). Importantly, depending on the underlying pathophysiological mechanisms, metacognitive judgments have different brain correlates. Indeed, the precuneus, a key hub of the default mode network that shows characteristic functional connectivity reduction in AD [21], is involved in incorrect metacognitive judgments [22]. On the other hand, impaired metacognitive abilities with a tendency towards underestimation in individuals with anxiety and depressive disorders are rather related to specific brain connectivity changes of the anterior prefrontal cortex, leading to cognitive dyscontrol [23]. These observations underline the possibility to discriminate SCD individuals with preclinical AD from others with psychiatric conditions, by comparing metacognitive abilities and corresponding connectivity features. Such distinctions might be used in memory clinics to identify individuals who are more likely to develop cognitive impairment and finally dementia (preclinical AD) from those with non-progressive forms of SCD (e.g., associated with psychiatric conditions). To the best of our knowledge, this issue has never been investigated so far.

The aim of this work is to describe the rationale and the study design of COSCODE.

## Methods

### Study design and participants

COSCODE is a 4-year observational, longitudinal, prospective, clinical-cohort study taking place at the Memory Center of the Geneva University Hospitals (GMC). The target sample consists of 120 individuals with SCD, 40 individuals living with MCI or dementia due to AD (i.e., positive control group), and 40 cognitively unimpaired volunteers (i.e., negative control group). Individuals living with SCD, MCI, and dementia will be recruited from the cohort of individuals diagnosed at the GMC. The negative control group will be recruited by an advertising campaign of the GMC. General inclusion criteria are the following: (i) age over 40 years, (ii) no major psychiatric disorders, (iii) no contraindications to MRI, (iv) no severe behavioural disturbances (e.g., ability to undergo the procedures of the study), (v) no severe diseases such as a malignant neoplasm within the last 5 years or other life threatening conditions, and (vi) no severe systemic diseases (e.g., renal failure, heart failure, decompensated diabetes).

Moreover, specific criteria will be adopted for each cognitive stage group (SCD, MCI, dementia). Specifically, SCD is defined based on the following criteria: persons asking for help and consulting to the GMC for self-experience of deterioration in cognitive abilities, without objective cognitive impairment detected through formal neuropsychological testing. MCI is defined based on the following clinical criteria: (i) objective evidence of cognitive impairment, (ii) cognitive concern reported by the patient and/or informant (family or close friend), and (iii) no functional impairment in daily living activities [24]. Individuals living with dementia are defined based on the same (i) and (ii) above criteria for MCI but differ from them for the impairment in the activities of daily living [25]. Alzheimer’s disease etiology will be defined based on the French National Alzheimer Database diagnostic model [26].

**Fig. 1:**
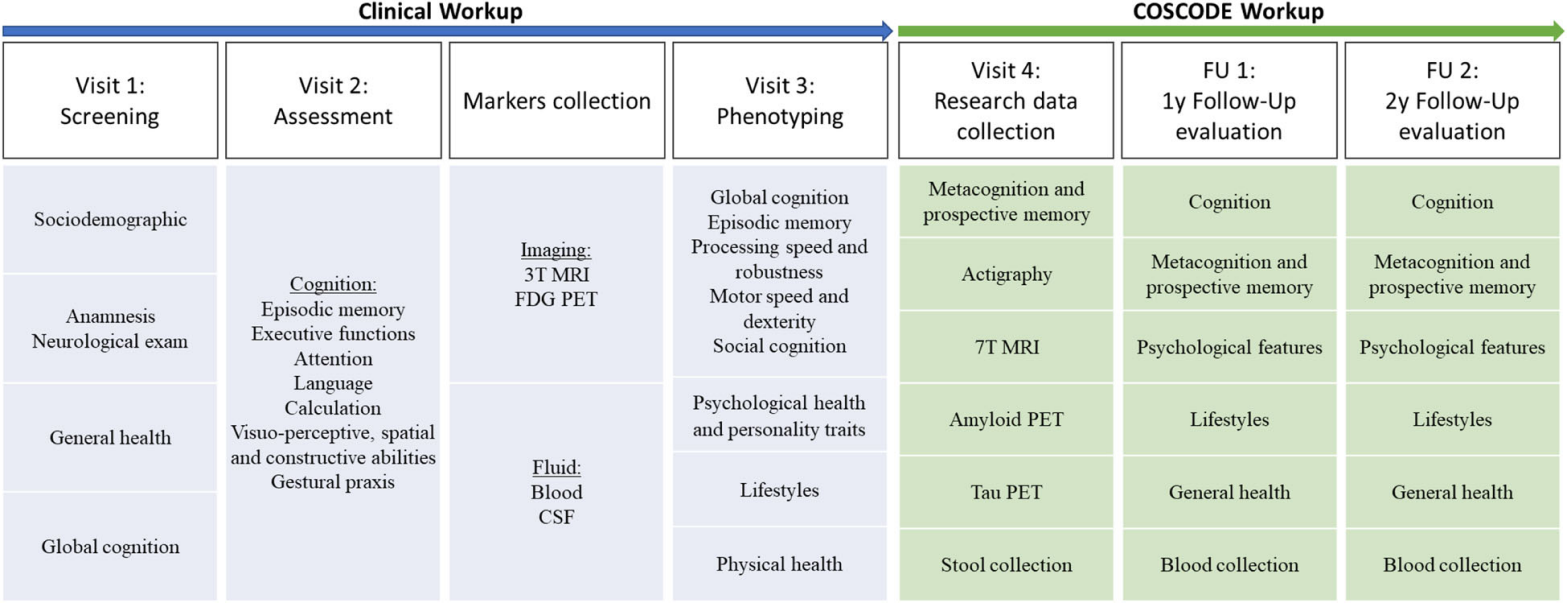
Clinical and research workups. The figure shows the clinical workup of the GMC and the research workup of COSCODE. During the first visit, sociodemographic features, general health, and global cognition (MMSE, Clock test, Three-Objects-Three-Places) are evaluated. In this visit, medical, family history, and medication information are collected, and a neurological exam is performed. Visit 2 consists of a complete neuropsychological assessment. Visit 3 includes scales and questionnaires on psychological health, lifestyles, and some additional cognitive tests. After Visit 3, the individual comes back to the GMC for a diagnostic restitution with a physician and a neuropsychologist. In this session, the COSCODE project will be proposed. During the COSCODE workup, the metacognition task, scales, and questionnaires on metacognition will be administered. Stool and saliva will be collected for all participants. 60 participants will undergo 7T MRI. Amyloid and tau PET, as well as actigraphy will be performed on around 60% of the sample. Behavioral follow-up at 1 and 2 years will be done for all participants

COSCODE participants will undergo standardized clinical procedures including (i) clinical and neurological assessment, (ii) neuropsychological testing, (iii) brain magnetic resonance imaging (MRI), and (iv) blood collection (Fig. 1). Additional procedures, such as amyloid positron emission tomography (PET), tau PET, fluorodeoxyglucose (FDG)-PET, and stool and saliva collection will be performed if deemed clinically useful, or in a context of other research projects. Moreover, 60 COSCODE participants (15 volunteers, 30 SCD, 15 individuals living with MCI or dementia) will undergo 7T MRI. Based on our retrospective data and ongoing research at the GMC, we expect to have stools and saliva samples for 95% of the participants, tau PET for 65%, amyloid PET for 50%, and FDG-PET for 30%. Each study participant will undergo annual behavioral follow-ups for 2 years as represented in Fig. 1.

The study was approved by the local ethics committee (CCER number: 2020-00403). All participants will sign an informed consent form.

### Primary and secondary endpoints

The primary endpoint is to test whether metacognition, sociodemographic, lifestyle, clinical and cognitive features, brain connectivity changes, and AD biomarkers can be used to discriminate different subgroups of SCD, based on both hypothesis-driven and data-driven approaches (Statistics section).

The secondary outcome is to assess clinical and cognitive trajectories of the different SCD subgroups.

### Data collection

In this section, we describe how the COSCODE data being used in the COSCODE analyses will be collected from diagnostic and research workups (see Fig. 1).

#### Clinical assessment

Clinical assessment will be performed by trained physicians conducting a semi-structured interview. This includes standard investigation of the following domains: (i) sociodemographics, (ii) medical and family history, (iii) medication, (iv) neurological exam, and (v) general health (Fig. 1).

#### Neuropsychological tests

An extensive psychometric test battery assessing global cognition, episodic memory, working memory, attention, language, calculation, praxis, executive functions, visuo-perceptive and spatial abilities, processing speed and robustness, motor speed and dexterity, and social cognition will be performed by expert neuropsychologists (Fig. 1, Table 1).

**Table 1:** Behavioral data collection

Behavioural assessment	
**Cognition**	
Global cognition	Mini-Mental State Examination, Montreal Cognitive Assessment, Clock test, Three-Objects-Three-Places
Episodic memory	Free and Cued Selective Reminding Test, Rey Auditory Verbal Learning Test, Logical Memory Story B, Doors test, Rey Osterrieth complex figure copy and recall
Working memory	Digit Span (WAIS-IV)
Attention	Coding (WAIS-IV), d2-R
Language	Naming (GREMOTs battery), writing (GREMOTs battery), reading comprehension (GREMOTs battery)
Calculation	Mental calculation, oral (Barcelona battery)
Praxis	Gestural praxis (BREP), constructional praxis (CERAD battery)
Executive functions	Semantic and Phonemic Fluency, Stroop Victoria, Trail-Making Test (GREFEX battery)
Visuo-perceptive and spatial abilities	Incomplete letters, dot counting, number location, cube analysis (VOSP battery)
Processing speed and robustness	Simple reaction time
Motor speed and dexterity	Groove Peg Board, tapping test (WPS electronic)
Social cognition	Facial emotion recognition (Mini-SEA battery)
**Metacognition and prospective memory**	
Knowledge	Motivation Assessment for Cognitive Activities Questionnaire, Metamemory in Adulthood Questionnaire, Metacognitive Prospective Memory Inventory
Monitoring	Metacognition task
**Psychological health**	
Personality traits	NEO - Personality Inventory – 3
Depression, anxiety and stress	Hospital Anxiety and Depression Scale, State-Trait Anxiety Inventory, Depression Anxiety Stress Scale
Ruminations, negative thoughts and worries	Ruminative response scale, Perseverative Thinking Questionnaire, Penn State Worry Questionnaire
Alexithymia	Toronto Alexithymia Scale-20
Cognitive coping strategies	Cognitive Emotion Regulation Questionnaire
Attachment style	Attachment scale, Experiences in Close Relationships-Revised Scale (short version)
**Lifestyles**	
Physical and leisure activities	Historical Leisure Activity Questionnaire, Physical Activity Scale
Social contact	Lubben Social Network Scale
Food habits	Food Frequency Questionnaire
Cognitive reserve	Cognitive Reserve Index Questionnaire
Quality of life	Euro-Quality of Life (EQ-5D), 12-Item Short Form Survey (version 2)
**General health**	
Activity of daily living	Amsterdam-instrumental activities daily living (short version)
Cognitive complaints	Cognitive Change Index self and informant
Comorbidities	Cumulative Illness Rating Scale (CIRS), Cardiovascular Risk Factors, Aging, and Incidence of Dementia (CAIDE)
Sleep	Pittsburgh Sleep Quality Index
Fatigue	Multidimensional Assessment of Fatigue Scale

#### Metacognition task

The metacognition paradigm is based on the traditional laboratory assessment of prospective memory (PM), with the PM task embedded in an ongoing lexical decision task (LDT) [27]. The paradigm was adapted from previous works [28, 29] to include metacognitive judgments.

Participants will be instructed to memorize a list of pairs of words and to carry out a specific action (press a specific key) when identifying the first word of a pair (PM cue) during the LDT. If they do so, participants will provide the second word of the respective word pair. This approach allows for examining the two different, prospective and retrospective, components within the PM performance (see [30] for a similar approach). The metacognitive judgements will be assessed through participants’ estimations of their probability of successful PM performance before and after each task. Two blocks with different lists of words pairs will be used, allowing for evaluation of changes in the learning strategies and related predictions.

#### Questionnaires and scales

Several questionnaires and scales have been selected to evaluate the following domains: personality traits, depression, anxiety and stress, ruminations, negative thoughts and worries, alexithymia, cognitive coping strategies, attachment style, physical and leisure activities, social contacts, eating habits, cognitive reserve, quality of life, activity of daily living, sleep, fatigue, metacognition, and prospective memory (knowledge and monitoring). Some of these questionnaires are self-administered and will be preferably performed digitally via REDCap (https://projectredcap.org/). Table 1 reports a detailed overview of the questionnaires. Since the project includes several questionnaires, we will use a brief survey to collect the feedback of the study participants on the questionnaires’ feasibility.

#### Assessment of subjective cognitive complaints

The SCD+ features will be assessed by a neuropsychologist or physician using a semi-structured interview during the first visit [1]. Moreover, the Cognitive Change Index (self and informant versions) will be used to assess the change of the cognitive function over the last 5 years.

#### MRI at 3T and 7T

3T MRI will be acquired using the following standard Alzheimer’s Disease Neuroimaging Initiative (ADNI) compatible sequences: T1 MPRAGE for brain atrophy evaluation and volumetry, 3D T2 FLAIR and T2 turbo spin echo (TSE) for vascular lesions assessment, SWI for identifying cerebral microbleeds and other hemorrhagic lesions, DTI for structural connectivity assessment, ASL for brain perfusion quantification, and resting-state functional MRI (rsfMRI) for functional connectivity assessment. 7T MRI will be acquired for 60 COSCODE participants and will include T1 MP2RAGE, SWI, multi-shell diffusion MRI, and rsfMRI.

The MRI exams will thus provide a very comprehensive set of biomarkers for group comparisons, namely gray matter volume, white matter lesions and stigmata of small-vessel disease, microstructural integrity, neuron density, intra-axonal injury, inflammation, and structural connectivity, functional connectivity, perfusion, and microbleeds (for a complete overview, see Table 2).

**Table 2:** Biomarker collection

Biomarkers	
**Imaging**	
**Molecular**	
Amyloid PET	Standardized uptake value ratio
Tau PET	Standardized uptake value ratio
Fluorodeoxyglucose PET	Standardized uptake value ratio
**Magnetic resonance**	
T1 3D MP-RAGE	Gray matter volume
3D FLAIR	White matter lesions volume
rsfMRI	Functional connectivity at rest
DTI	Mean diffusivity, radial diffusivity, fractional anisotropy, axonal density, intra-axonal injury, inflammation
SWI	Number of Microbleeds
ASL	Gray matter perfusion indexes
**FLUID**	
CSF	Aβ40, Aβ42, P-tau, T-tau
Blood	Aβ40, Aβ42, P-tau181, P-tau217, NfL, APOE, inflammatory markers
Stool and saliva	Gut microbiota composition

#### PET acquisition

Different PET tracers are used in clinical routine or related research projects assessing glucose metabolism (F 18, fluorodeoxyglucose), amyloid (18F-florbetapir, 18F-flutemetamol, 18F-florbetaben), and tau (18F-flortaucipir). The PET/CT scans will be acquired at the division of nuclear medicine and molecular imaging of the Geneva University Hospitals using either a Siemens Biograph mCT or a Vision PET/CT scanner (both Siemens, Erlangen, Germany).

#### Cerebrospinal fluid (CSF)

CSF will be collected only for participants who agree to undergo a lumbar puncture. 20mL of CSF will be collected and analyzed with quantification of Aβ, P-tau, and T-tau levels. With a usual CSF production rate 0.3–0.4mL/min it will take 50–70 min to replace the withdrawn amount. Collection and handling of CSF samples will be performed according to the Geneva University Hospitals internal protocols and stored at the institution’s own biobank.

#### Blood

The blood (around 47mL) will be collected at the GMC. Eight aliquots of serum, 8 containing EDTA, and 1 with whole blood, 4 PaxGene RNA tubes will be stored at the biobank of the Geneva University Hospitals. EDTA tubes for APOE genotyping will be stored at −80°C. A real-time TaqMan assay (Applied Biosystems) will be performed to test for DNA integrity and quality assessment by electrophoresis. APOE genotyping will be performed automatically by the same instrument and verified by visual inspection of the generated fluorescence plots. A 1 mL of frozen plasma will be sent to the Department of Psychiatry and Neurochemistry at the Institute of Neuroscience and Physiology, Göteborg, Sweden, where blood-based biomarkers (P-tau181, P-tau217, NfL, and Aβ) will be extracted.

#### Microbiota

Stool samples will be collected at home by the participants using special containers, then transferred to the GMC by the study participants or a proxy. Aliquots will be sent on the same day of blood withdrawal to the Genomic Research Lab at Geneva University Hospitals and/or will be stored in the Department of Genetic Medicine or in a secured freezer in the GMC. The saliva will be collected at the GMC and will be stored together with the stool samples. Analyzing these biological materials, we will obtain the gut and saliva microbiota compositions.

#### Physical health assessment

The physical exam will be performed automatically using a TANITA MC-780MA P. Body composition is automatically provided through several measures including weight, body fat percentage, body water percentage, muscle mass, metabolic age, bone mass, and visceral fat.

Physical activity and sleep will be objectively recorded using a medical-grade wearable device, ActiGraph GT9X (ActiGraph, Pensacola, FL, USA) for 24h, 7 days (30 Hz recording) on the non-dominant wrist.

### Data storing

Source data from standard care visits will be collected in the study participant’s medical records. Documented medical histories and narrative statements relative to the individual’s state and evolution during the study will be maintained in the institutional (Geneva University Hospitals) electronic patient medical record system. All other experimental non-medically relevant, study-specific data will be inscribed in an electronic case report form (eCRF). Study data will be stored in a digital archive developed on RedCap (http://project-redcap.org). Data will be made available upon reasonable request by the principal investigator.

### Statistics

The main analysis will include a hypothesis-driven and a data-driven analyses to identify and further validate the existence of distinct subgroups of SCD.

As a first step, we will assign each individual with SCD to one of 4 main a-priori defined clinical groups which represent different possible etiologies of the cognitive complaints identified based on an analysis of retrospective clinical reports: neurological comorbidities (brain injury, transient ischemic attacks, cerebrovascular disease), physical comorbidities (cardiovascular risk factors such as hypertension or diabetes, physical disability), psychopathology (neuroticism, subtle depression or anxiety, or other personality traits), and unknown (no identifiable reason associated to the complaints) [31]. Then, we will compare metacognition, sociodemographic, lifestyle, clinical and cognitive features, brain connectivity changes, and AD biomarkers features among them.

As a second step, we will apply a data-driven approach to extract subgroups of SCD without a priori hypothesis. Indeed, a cluster analysis for mixed data type will be performed using all variables available. In particular, metacognition, sociodemographic, lifestyle, clinical and cognitive features, brain connectivity changes, and AD biomarkers.

Finally, to assess for the differences between groups in the longitudinal clinical and cognitive trajectories, a linear mixed model with all available time-points will be used. Possible covariates will be sociodemographic, clinical, cognitive and metacognitive, psychiatric, lifestyle, and biomarkers.

## Expected results

A previous work using a hypothesis-driven analysis on retrospective data identified 4 SCD subgroups [31]. However, the data used were only clinical. Therefore, adding metacognition, connectivity, and the other biomarkers, we will expect a more accurate phenotyping. We expect to find at least 3 SCD subgroups: AD-like, psychopathology-like, and one unknown group. The AD-like group should be characterized by the presence of AD biomarkers, metacognition deficits towards overestimation, and AD-like connectivity profile (changes in network connectivity within the default mode network) while the psychopathology-like group should include individuals with negative AD biomarkers, metacognition deficits towards underestimation, and connectivity changes in frontal circuits. We hypothesize that the data-driven approach will confirm this grouping and the differences among groups.

## Discussion

### Impact

The COSCODE protocol was extensively harmonized with other European SCD initiatives (DELCODE, SCIENCE, INSIGHT preAD) and represents another step forward through a standardized operationalization and assessment of the concept of SCD. All these projects are trying to identify the progression according to pathophysiological biomarkers status such as amyloid, tau, and neurodegeneration. However, COSCODE proposes an alternative approach, aiming to clinically define the constellation of signs and symptoms associated with different clinically meaningful subtypes of SCD. This evidence will be used by clinicians to better manage SCD individuals and provide them with clear answers, with subsequent referral to prevention interventions appropriate for each specific SCD subtype.

COSCODE will allow to (i) define the constellation of signs and symptoms associated with different etiologies of SCD; (ii) define pharmacological and non-pharmacological, as well as connectivity-based interventions aiming to delay neurodegeneration and mitigate memory complaints; and (iii) clarify the taxonomy of SCD.

### Limitation

One of the main limitations of the present study is the lack of availability of PET or CSF biomarkers for all participants. Indeed, the clinical nature of this study does not allow to perform amyloid and tau PET and/or CSF in all participants because some of these procedures are not implemented in clinical practice. However, we will overcome this limitation by collecting blood-based biomarkers.

Moreover, since it is a novel paradigm, we were unable to do a power analysis and properly estimated the sample size. Nevertheless, this project has allowed us to modify our clinical practice at the Geneva Memory Center, giving us the opportunity to define a protocol to deeply phenotype the SCD individuals in the next years. Therefore, we will be able to collect more data than those originally planned by the COSCODE project (around 400 new individuals per year ranging from SCD to dementia).

Another limitation is the duration of the follow-up. INSIGHT-preAD study has shown that a 30-month follow-up could not be enough to see a decline in SCD study participants. Indeed, only 2% progressed to prodromal AD in 30 months [19]. However, individuals at high risk of dementia will be followed up in clinical practice even after the 24 months of the study.

## Conclusion

The COSCODE project will allow us to identify potential etiologies of the SCD and their evolution over time to finally provide meaningful answers to SCD individuals. Its results will pave the way to the development of cost-effective risk profiling and prevention protocols, that will ultimately lead to a more rational use of health care resources.

## Data Availability

Not applicable.
